# HS-GC/MS-Based Targeted Blood VOC Panel for Canine Cancer Screening Using an Integrated Classification Framework in Retrospective Korea and Thailand Cohorts

**DOI:** 10.3390/ijms27188016

**Published:** 2026-09-09

**Authors:** Kyung-Geun Ahn, Jin-Wook Chris Kim, Soon Chul Gwak, Eunkyung Oh, Seyeon Lee, Jedsada Siripoonsub, Sirintra Sirivisoot, Patharakrit Teewasutrakul, Somporn Techangamsuwan, Suparat Chaipipat, Kannika Siripattarapravat, Manakorn Sukmak, Anudep Rungsipipat, Giup Jang, Geon A Kim

**Affiliations:** 1 Center for Veterinary Bioinformatics Research and Development, MetaDx Inc., Hanam 12939, Republic of Korea; 2 Department of Biomedical Laboratory Science, University of Health Science, Eulji University, Uijeongbu 11759, Republic of Korea; 3 MetaDx Inc., Seoul 06248, Republic of Korea; 4 Center of Excellence for Companion Animal Cancer, Department of Pathology, Faculty of Veterinary Science, Chulalongkorn University, Bangkok 10400, Thailand; bongbank.jeds@gmail.com (J.S.); anudep.r@chula.ac.th (A.R.); 5 Oncology Clinic, Small Animal Teaching Hospital, Faculty of Veterinary Science, Chulalongkorn University, Bangkok 10400, Thailand; 6 Department of Pathology, Faculty of Veterinary Science, Chulalongkorn University, Bangkok 10400, Thailand; somporn.t@chula.ac.th; 7 Kasetsart University Veterinary Diagnostic Laboratory—Bangkhen, Faculty of Veterinary Medicine, Kasetsart University, Bangkok 10900, Thailand; suparat.ch@ku.th (S.C.); kannika.si@ku.th (K.S.); 8 Department of Pathology, Faculty of Veterinary Medicine, Kasetsart University, Bangkok 10900, Thailand; 9 Department of Farm Resources and Production Medicine, Faculty of Veterinary Medicine, Kasetsart University, Kamphaeng Saen Campus, Nakhon Pathom 73140, Thailand

**Keywords:** VOCs, Metabolomics, HS-GC/MS, Multicenter Study, Veterinary Medicine, cancer screening

## Abstract

Canine cancer screening has an important limitation in that organ-specific screening strategies used in human medicine cannot be directly applied across routine veterinary clinical practice. Blood-based volatile organic compound (VOC) profiling may provide a practical approach for pan-cancer screening; however, its application in multicenter and multinational settings requires evaluation that accounts for cohort heterogeneity and analytical variability. In this study, we evaluated the canine cancer screening performance of an integrated classification framework using a headspace gas chromatography–mass spectrometry (HS-GC/MS)-based targeted blood VOC panel in a retrospective multicohort dataset comprising dogs recruited in Korea and Thailand. Whole-blood samples from 590 dogs recruited in Korea and Thailand were analyzed, including 167 dogs in the malignant tumor group and 423 dogs in the non-cancer control group. The predefined targeted VOC panel consisted of aromatic compounds and straight-chain alkanes and was analyzed using HS-GC/MS. The Korea and Thailand cohorts were incorporated into a single integrated classification framework rather than being separated into country-specific models. To evaluate performance variability associated with data partitioning, cohort-aware stratified training–validation splitting was repeated 30 times within the integrated retrospective cohort, with both national cohorts represented in the training and validation sets in each repeat. Class imbalance correction was applied only to the training data, and validation performance was assessed using both threshold-dependent and threshold-independent metrics. Across the 30 repeated validations, the integrated framework achieved an AUROC of 0.926 ± 0.019 and an AUPRC of 0.894 ± 0.023. Balanced accuracy, accuracy, sensitivity, specificity, F1-score, MCC, and Cohen’s κ were 0.858 ± 0.024, 0.895 ± 0.018, 0.772 ± 0.048, 0.943 ± 0.022, 0.805 ± 0.033, 0.736 ± 0.046, and 0.734 ± 0.045, respectively. In the sample-level prediction score analysis, the median predicted cancer probabilities for cancer and non-cancer samples were 0.954 and 0.041, respectively, indicating clear probability-based separation between the two groups. In the cohort-specific analysis, the discrimination signal was maintained in both the Korea and Thailand cohorts, although threshold-dependent performance differed between the two cohorts. These findings indicate that an HS-GC/MS-based targeted blood VOC panel provided a consistent cancer-associated classification signal under repeated internal validation within the specific integrated retrospective dataset evaluated in this study. The results demonstrate the feasibility of evaluating a common classification framework across the Korea and Thailand cohorts without constructing separate country-specific models, while not establishing independent external generalizability or platform-independent robustness. Because the Korea and Thailand cohorts were completely confounded with the Agilent and Thermo Fisher analytical platforms, respectively, the independent contributions of cohort characteristics and analytical platform cannot be determined from the present data. Further independent prospective validation in newly recruited populations and institutions, together with disease-control evaluation including clinically relevant controls, cohort-specific calibration, evaluation across analytical platforms, and assessment of clinical utility within real-world workflows, is required before the external generalizability and clinical applicability of the framework can be determined.

## 1. Introduction

Various cancers in dogs share histological and clinical characteristics, including signs and symptoms, that closely resemble those of human cancers [1]. However, cancer screening in dogs presents challenges that differ fundamentally from those in human medicine. In human medicine, targeted screening tests for specific cancer types, such as mammography, colonoscopy, and prostate-specific antigen (PSA) testing, have been standardized [2,3,4,5]. In contrast, applying such tests individually across multiple major cancer types in dogs may not be practical because of the potential need for anesthesia, limited accessibility, financial burden, and owner compliance. In addition, because dogs cannot verbally express symptoms, cancer may remain undetected until clinical signs are recognized by the owner or veterinarian, and detection may be delayed during asymptomatic or nonspecific symptomatic stages [6]. Although evidence is limited, one cohort study reported that the rate of newly detected cancers through health screening, including complete blood count, serum biochemistry panel, and urinalysis, was only 4% [7]. These characteristics indicate the need for a reliable pan-cancer screening approach capable of evaluating multiple cancer types simultaneously in dogs.

Tumor tissues may undergo characteristic metabolic reprogramming, including altered energy metabolism associated with the Warburg effect and increased cell membrane turnover [8]. During this process, various volatile metabolites, including aromatic compounds and low-molecular-weight hydrocarbons, may be generated or undergo changes in their distribution. These compounds can be released into blood or breath, forming cancer-associated volatile organic compound (VOC) profiles. Accordingly, VOC analysis has gained attention as a noninvasive or minimally invasive approach for cancer screening [9].

VOC-based cancer screening is considered an approach with potential clinical applicability. However, in conventional untargeted volatilomics approaches, ensuring the selectivity and reproducibility of the analytes has remained an important limitation. In particular, systemic inflammation can promote lipid peroxidation through reactive oxygen species (ROS), resulting in increased levels of various aldehyde VOCs, including hexanal [10]. These compounds may also be elevated in benign proliferative diseases or non-neoplastic inflammatory diseases and may therefore be influenced by factors other than tumors [11]. If these compounds are overrepresented in untargeted analyses, the relative discriminative power of cancer-associated VOC signals may be reduced [10,12]. Therefore, VOC-based cancer screening approaches require a strategy that considers not only the exploration of a large number of VOCs but also the selectivity and reproducibility of the analytes and the potential for signal variation caused by non-neoplastic factors.

In multicenter clinical studies, non-biological variation may arise from differences in sample collection, storage, transportation, analytical instruments, laboratory environments, and operational conditions [13]. In particular, when data are generated using GC-MS platforms from different manufacturers, the absolute values of chromatographic peak areas and the characteristics of signal distributions may differ. Such variation can impair the generalization performance of classification models. Therefore, establishing a VOC-based screening system applicable to multinational and multicenter settings requires a practical analytical and validation strategy that accounts for both biological heterogeneity and non-biological analytical variability.

CancerVET^®^ (MetaDx Inc., Seoul, Republic of Korea) is an HS-GC/MS-based targeted VOC panel analysis system developed on the basis of these clinical needs and the biological rationale for VOC-based cancer screening. This approach performs targeted analysis of predefined VOCs in whole-blood samples [14] and is designed to classify cancer and non-cancer samples through integrated interpretation of these signals. The focus of this study was not to determine the biological directionality of individual VOC markers or to conduct novel biomarker discovery, but to evaluate whether an integrated classification framework using a predefined targeted VOC panel demonstrates stable screening/classification performance in a multinational cohort setting.

For a new screening framework to be widely implemented in real-world clinical settings, internal performance derived during the development stage alone is insufficient. It is necessary to evaluate whether performance is maintained across different cohort compositions and validation conditions [15,16]. In particular, companion animal clinical testing environments may vary across countries in terms of breed composition, living environment, dietary characteristics, and clinical practice settings. Therefore, whether a screening model developed in a single country or a single cohort performs consistently in populations from other regions is an important consideration for international expansion [17].

However, global applicability cannot be sufficiently evaluated solely by applying a model developed in one country to a cohort from another country. When biological and environmental heterogeneity exists across countries, a model optimized for a specific country may show reduced performance or calibration differences in multinational settings, potentially leading to the inefficiency of developing separate algorithms for each country [18]. Therefore, to meaningfully evaluate international applicability, it is necessary to integrate cohorts recruited from different countries and assess whether a single integrated framework can maintain consistent screening performance under the same methodological approach [19,20]. This approach represents a key step in evaluating the feasibility of implementing a multinational screening system that can function as a common platform without developing separate country-specific models.

In this study, we integrated canine cohorts recruited in Korea and Thailand and evaluated the classification performance of a targeted VOC panel-based integrated classification framework for discriminating cancer from non-cancer samples. Rather than evaluating the two national datasets as separate country-specific models, this study was designed to include each national cohort in both the training and validation sets through cohort-aware data partitioning and to assess classification performance within a single integrated framework. This design evaluated the feasibility of a common classification framework applicable to multinational cohorts without constructing separate country-specific models. In addition, a repeated cohort-aware internal validation strategy was applied to evaluate both the average performance of the integrated framework and performance variability associated with data partitioning within the integrated retrospective cohort, rather than relying on a single data split. Because the detailed data processing procedures and implementation structure of the prediction algorithm included in the CancerVET®-based analytical system constitute proprietary technology, they were not disclosed. Instead, this study focused on classification performance, repeated internal validation stability, and cohort-specific performance when applying an integrated framework without separating country-specific models.

## 2. Results

### 2.1. Study Cohort Composition and Repeated Validation Dataset

This study included a total of 590 canine samples collected in Korea and Thailand. The overall cohort consisted of 423 non-cancer samples and 167 cancer samples. By country, the Korea cohort included 321 samples (non-cancer, n = 232; cancer, n = 89), and the Thailand cohort included 269 samples (non-cancer, n = 191; cancer, n = 78). In this analysis, data from the two countries were evaluated within a single integrated framework rather than being separated into country-specific models. The overall cohort and class distribution are summarized in Table 1.

Age and sex/neuter status metadata were available for all 590 dogs, and the baseline characteristics are summarized in Supplementary Table S2. In the overall cohort, the mean age was 8.1 ± 4.4 years, with a median of 8.0 years. Cancer samples were older than non-cancer samples, with mean ages of 10.6 ± 3.4 and 7.2 ± 4.3 years, respectively. Age distribution also differed between the national cohorts; the mean age was higher in the Korea cohort (8.7 ± 4.5 years) than in the Thailand cohort (7.4 ± 4.2 years). Biological sex distribution was similar between cancer and non-cancer samples, whereas sex/neuter status distribution differed across class and cohort strata. In particular, the proportion of neutered or spayed dogs was higher in the cancer group than in the non-cancer group and was also higher in the Korea cohort than in the Thailand cohort. These findings indicate that heterogeneity in baseline characteristics was present within the integrated multinational cohort and should be interpreted as part of cohort characterization rather than as evidence that age or sex/neuter status influenced classification performance.

Internal repeated validation was performed using 30 random seeds within the integrated retrospective cohort. In each repeat, the validation set consisted of 178 samples, including 50 cancer samples and 128 non-cancer samples. By country, the validation set consisted of 97 samples from the Korea cohort (cancer, n = 27; non-cancer, n = 70) and 81 samples from the Thailand cohort (cancer, n = 23; non-cancer, n = 58). Thus, each repeated validation run was designed to evaluate performance variability across different random seeds while maintaining the same class and cohort composition.

To further evaluate whether the observed classification performance could be substantially explained by the age difference between the cancer and non-cancer groups, an additional sensitivity analysis was performed in dogs aged ≥8 years. The age-restricted cohort included 336 dogs, comprising 136 cancer samples and 200 non-cancer samples. Using the same repeated internal validation framework described for the primary analysis, the age-restricted analysis yielded a mean AUROC of 0.911 ± 0.029 and a mean AUPRC of 0.909 ± 0.027. At the predefined probability threshold of 0.5, sensitivity was 0.763 ± 0.055 and specificity was 0.925 ± 0.038. The corresponding 95 % confidence intervals were 0.900 – 0.921 for AUROC, 0.899 – 0.920 for AUPRC, 0.743 – 0.784 for sensitivity, and 0.910 – 0.939 for specificity. Detailed performance estimates are provided in Supplementary Table S3. The age-restricted analysis was considered a supportive sensitivity analysis because the restriction to dogs aged ≥8 years reduced the available sample size. Therefore, the results do not establish that age has no independent contribution to the classification signal; rather, they provide an additional assessment of whether the observed classification performance persists when the comparison is restricted to dogs aged ≥8 years.

### 2.2. Repeated Validation Performance of the Integrated Classification Framework

**Figure 1:**
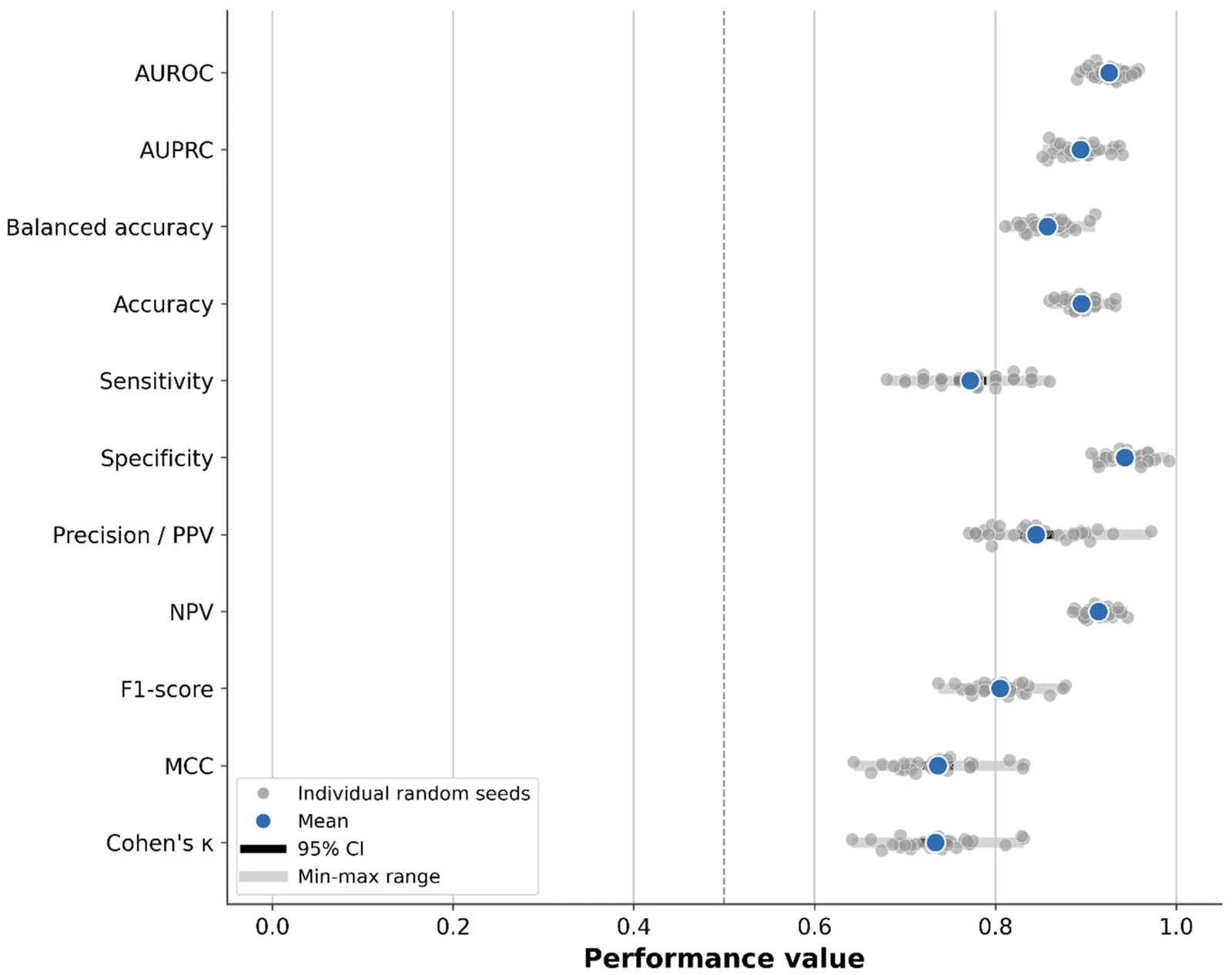
Repeated internal validation performance across 30 random seeds within the integrated retrospective cohort. Performance metrics of the integrated targeted VOC panel-based classification framework were evaluated across 30 repeated internal validation runs using different random seeds within the integrated retrospective cohort. Each gray point represents the metric value from an individual validation run, the blue point indicates the mean value across runs, the black horizontal bar denotes the 95% confidence interval of the mean, and the light gray bar denotes the min–max range. Performance was assessed using AUROC, AUPRC, balanced accuracy, accuracy, sensitivity, specificity, positive predictive value (PPV), negative predictive value (NPV), F1-score, Matthews correlation coefficient (MCC), and Cohen’s κ. Class assignment was based on a predicted cancer probability threshold of 0.5, with cancer defined as the positive class.

The performance of the integrated targeted VOC panel-based classification framework was evaluated across 30 repeated internal validation runs within the integrated retrospective cohort (Figure 1), and the mean, SD, 95 % CI, and min–max range of the major performance metrics are presented in Table 1. Across the repeated validations, the AUROC was 0.926 ± 0.019, with a 95% CI of 0.919–0.933 and a seed-level range of 0.890–0.958. The AUPRC was 0.894 ± 0.023, with a 95% CI of 0.885–0.903 and a seed-level range of 0.852-0.940. These results indicate that the discrimination metrics were maintained within a relatively narrow range across repeated evaluations and were not strongly dependent on a single split or a specific random seed.

Threshold-dependent classification performance was evaluated using a predicted cancer probability threshold of 0.5. Balanced accuracy was 0.858 ± 0.024 (95% CI, 0.849–0.866), and overall accuracy was 0.895 ± 0.018 (95% CI, 0.888–0.902). When cancer was defined as the positive class, sensitivity was 0.772 ± 0.048 (95% CI, 0.754–0.790), and specificity was 0.943 ± 0.022 (95% CI, 0.935–0.951).

Relatively consistent performance was also observed for predictive value metrics. PPV was 0.845 ± 0.052 (95% CI, 0.825–0.864), and NPV was 0.914 ± 0.016 (95% CI, 0.908–0.920). The F1-score was 0.805 ± 0.033 (95% CI, 0.793–0.818). In addition, MCC and Cohen’s κ, which complementarily account for class imbalance and chance agreement, were 0.736 ± 0.046 (95% CI, 0.719–0.754) and 0.734 ± 0.045 (95% CI, 0.717–0.751), respectively.

### 2.3. ROC and Precision–Recall Curve Analysis

**Figure 2:**
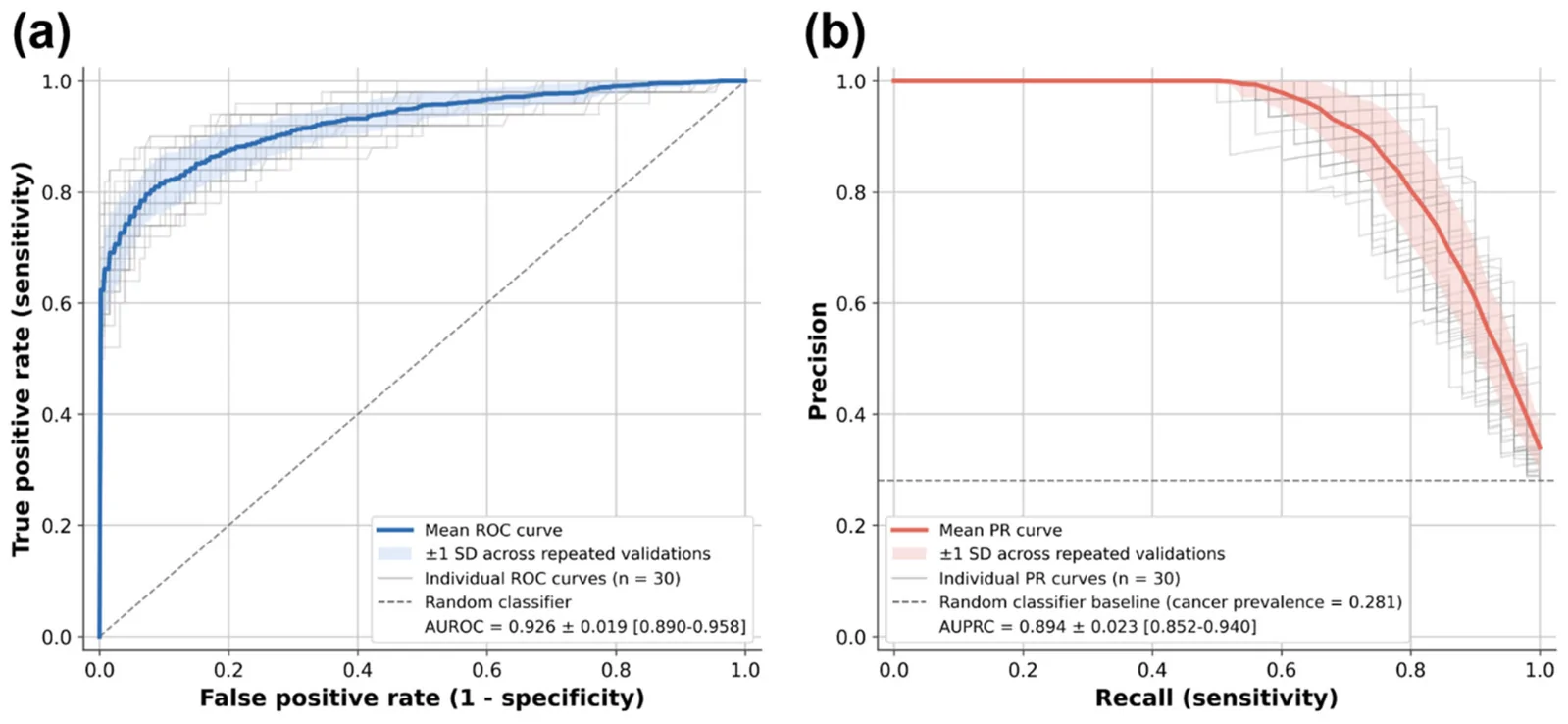
Mean ROC and precision–recall curves across repeated internal validation runs. Receiver operating characteristic (ROC) and precision–recall (PR) curves were generated across 30 repeated internal validation runs within the integrated retrospective cohort. (**a**) ROC curve analysis. Thin gray lines represent individual seed-level ROC curves, the solid blue line represents the mean ROC curve, and the shaded area indicates ±1 standard deviation across repeated validations. The dashed diagonal line indicates the performance of a random classifier. (**b**) PR curve analysis. Thin gray lines represent individual seed-level PR curves, the solid red line represents the mean PR curve, and the shaded area indicates ±1 standard deviation across repeated validations. The dashed horizontal line indicates the random-classifier baseline corresponding to the prevalence of cancer samples in the repeated validation sets. AUROC and AUPRC summaries are reported as mean ± standard deviation with min–max ranges across repeated validations.

To evaluate threshold-independent discrimination performance across the repeated internal validation runs, seed-level ROC curves and precision–recall (PR) curves were generated (Figure 2). In the ROC curve analysis, individual seed-level curves were generally distributed in the upper-left region, and the mean AUROC was 0.926 (Figure 2a). The seed-level standard deviation of AUROC was 0.019, and the min–max range was 0.890–0.958, indicating limited variability in discrimination performance across repeated validations.

In the PR curve analysis, the mean AUPRC was 0.894, which was substantially higher than the random-classifier baseline of 0.281, corresponding to the cancer prevalence in the validation set (Figure 2b). Because the PR curve directly reflects the precision–recall trade-off for the positive class, these results indicate that classification performance for the cancer class was not explained simply by class distribution. Instead, they reflect the tendency of the predicted cancer probability to assign cancer samples preferentially to higher-score regions. The seed-level range of AUPRC was 0.852–0.940, indicating that PR-based performance was also maintained across the repeated validations.

Taken together, the ROC and PR curve results show that the integrated targeted VOC panel-based classification framework maintained both threshold-independent discrimination performance and positive-class enrichment performance under the repeated internal validation setting. In particular, the high AUROC and AUPRC values indicate that both overall ranking performance between non-cancer and cancer samples and precision–recall performance for cancer samples were preserved in the presence of class imbalance.

### 2.4. Sample-Level Prediction Score Distribution

**Figure 3:**
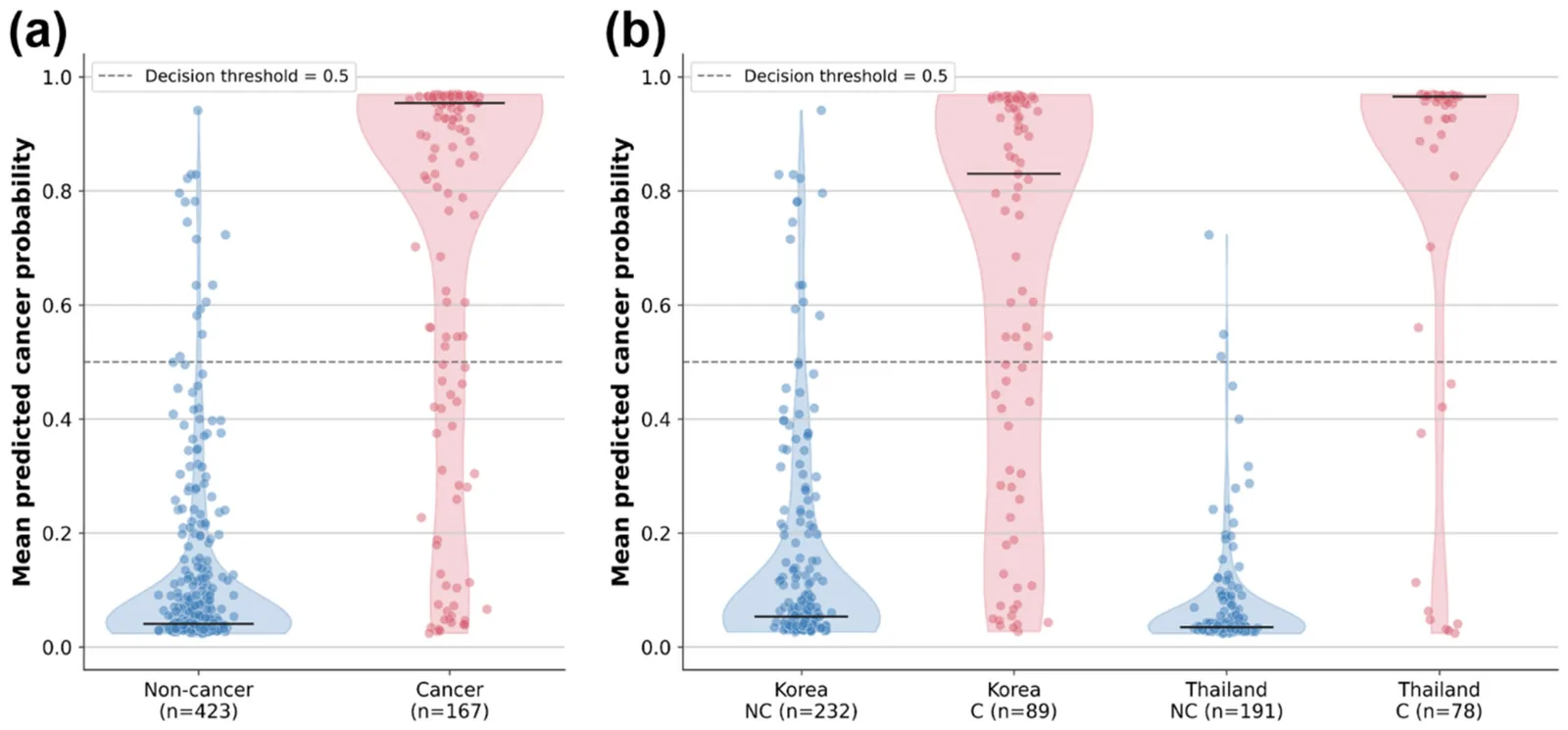
Prediction score distributions at the sample level. Prediction score distributions of the integrated targeted VOC panel-based classification framework are presented as sample-level mean predicted cancer probabilities. Because individual samples could appear in validation sets across multiple repeated runs, predicted cancer probabilities were aggregated at the sample level by averaging values across validation occurrences. (**a**) Overall distribution of predicted cancer probabilities for non-cancer and cancer samples. (**b**) Cohort-specific distributions for Korea non-cancer, Korea cancer, Thailand non-cancer, and Thailand cancer samples. Violin plots represent the distribution of sample-level mean predicted cancer probabilities, and superimposed points represent individual samples. Horizontal black lines indicate group medians. The dashed horizontal line indicates the class assignment threshold of 0.5.

The sample-level distribution of predicted cancer probabilities derived from repeated internal validation was evaluated (Figure 3). Because the same sample could be included in the validation set across multiple validation runs, the predicted cancer probabilities across validation occurrences were averaged for each sample to calculate the sample-level mean predicted cancer probability. This approach was applied to account for repeated observations of the same sample under the repeated validation structure and to evaluate score separation at the individual sample level.

In the overall sample-level distribution, the mean predicted cancer probability of cancer samples was clearly higher than that of non-cancer samples (Figure 3a). For cancer samples, the sample-level mean predicted cancer probability had a mean of 0.745, a median of 0.954, and an interquartile range (IQR) of 0.545–0.966. In contrast, for non-cancer samples, the mean predicted cancer probability had a mean of 0.108, a median of 0.041, and an IQR of 0.033–0.091. These distributions indicate score-level separation, with cancer samples generally concentrated in higher-probability regions and non-cancer samples concentrated in lower-probability regions.

However, score overlap between the two classes was also observed. Based on the sample-level mean predicted cancer probability, 128 of 167 cancer samples were located at or above the 0.5 threshold, whereas 39 were below the threshold. Among 423 non-cancer samples, 406 were below the threshold, whereas 17 were at or above the threshold. This overlap is consistent with the occurrence of false-negative and false-positive results in threshold-based classification and represents an important finding, given that this framework is a probability-based screening/classification approach.

Class-associated separation was also observed in the cohort-specific score distributions (Figure 3b). In the Korea cohort, the mean predicted cancer probability of cancer samples had a mean of 0.656, a median of 0.830, and an IQR of 0.388–0.961, whereas that of non-cancer samples had a mean of 0.142, a median of 0.053, and an IQR of 0.037–0.141. In the Thailand cohort, the mean predicted cancer probability of cancer samples had a mean of 0.847, a median of 0.965, and an IQR of 0.934–0.967, whereas that of non-cancer samples had a mean of 0.066, a median of 0.035, and an IQR of 0.030–0.051. In both cohorts, the predicted probabilities of cancer samples were shifted toward higher values than those of non-cancer samples. However, in the Korea cohort, the lower quartile of the cancer score distribution was relatively lower, and the distribution was wider. This finding is consistent with the differences in sensitivity and F1-score observed in the subsequent cohort-specific performance analysis.

### 2.5. Cohort-Specific Performance in Korea and Thailand

**Figure 4:**
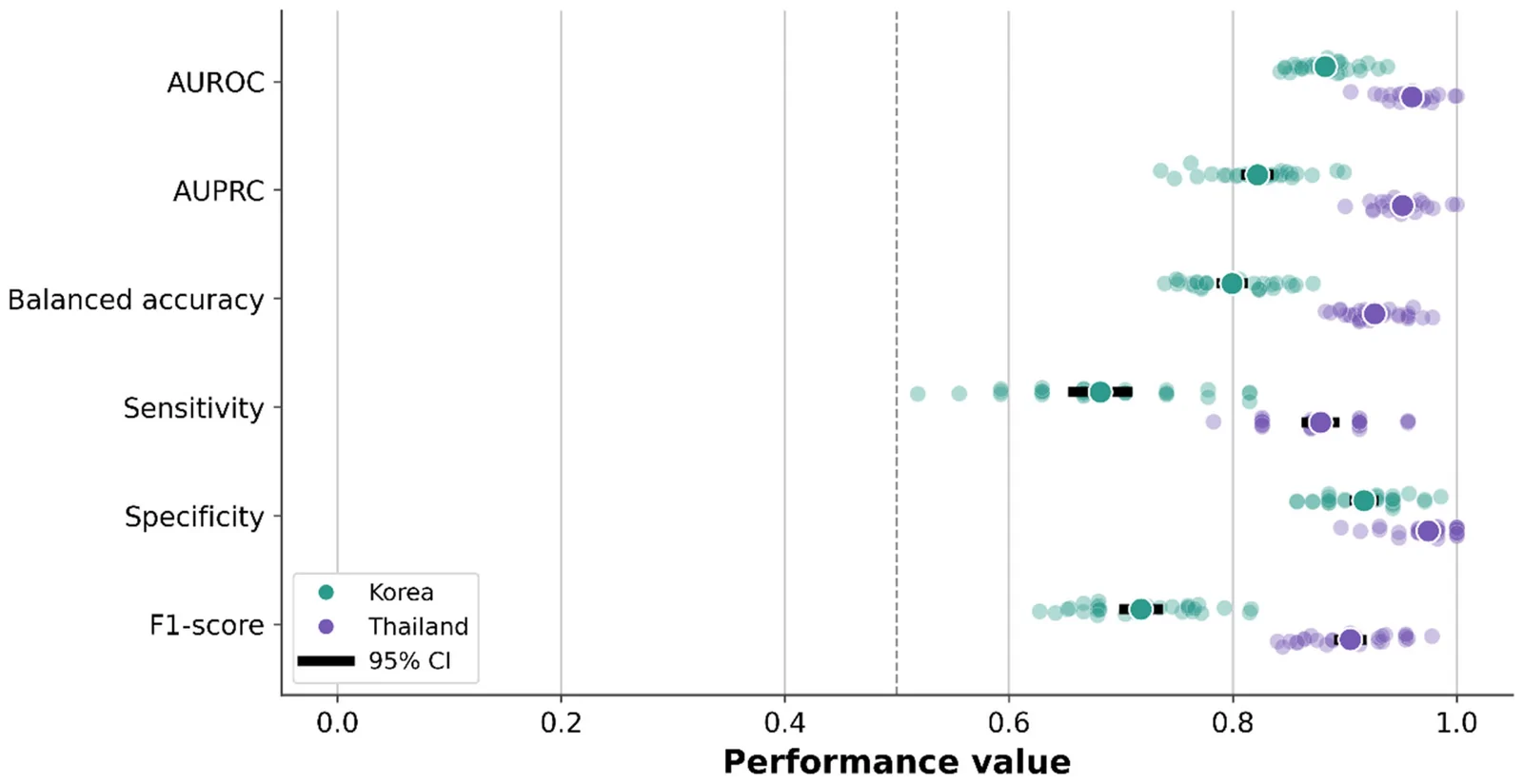
Cohort-specific performance across repeated internal validation runs. Performance of the integrated targeted VOC panel-based classification framework was evaluated separately in the Korea and Thailand cohorts across 30 repeated validation runs. Each point represents the metric value from an individual validation run within the corresponding cohort, and the larger colored point indicates the mean value across runs. Black horizontal bars indicate the 95% confidence interval of the mean. Metrics shown include AUROC, AUPRC, balanced accuracy, sensitivity, specificity, and F1-score. Class assignment was based on a predicted cancer probability threshold of 0.5, with cancer defined as the positive class.

To evaluate the integrated framework within each national cohort, repeated internal validation results were stratified by the Korea and Thailand cohorts (Figure 4). In the Korea cohort, AUROC was 0.882 ± 0.025 (95% CI, 0.873–0.892), and AUPRC was 0.822 ± 0.038 (95% CI, 0.808–0.836). Balanced accuracy was 0.799 ± 0.037 (95% CI, 0.786–0.813), sensitivity was 0.681 ± 0.076 (95% CI, 0.653–0.710), specificity was 0.917 ± 0.035 (95% CI, 0.904–0.930), and F1-score was 0.718 ± 0.053 (95% CI, 0.698–0.738).

In the Thailand cohort, AUROC was 0.960 ± 0.019 (95% CI, 0.953–0.967), AUPRC was 0.951 ± 0.021 (95% CI, 0.944–0.959), balanced accuracy was 0.926 ± 0.026 (95% CI, 0.917–0.936), sensitivity was 0.878 ± 0.046 (95% CI, 0.861–0.896), specificity was 0.975 ± 0.028 (95% CI, 0.964–0.985), and F1-score was 0.905 ± 0.039 (95% CI, 0.890–0.919). Thus, AUROC and AUPRC remained high in both cohorts, whereas threshold-dependent performance, particularly sensitivity and F1-score, was lower in the Korea cohort.

Compared with the Korea cohort, the Thailand cohort showed higher AUROC, AUPRC, balanced accuracy, sensitivity, specificity, and F1-score by approximately 0.078, 0.129, 0.127, 0.197, 0.058, and 0.187, respectively. These differences are consistent with the wider and lower-shifted cancer score distribution in the Korea cohort shown in Figure 4. This cohort-specific comparison was descriptive and was not intended to assess the superiority of country-specific models. Because the Korea and Thailand cohorts were completely confounded with the Agilent and Thermo Fisher analytical platforms, respectively, the observed differences in cohort-specific performance cannot be attributed independently to cohort characteristics or analytical platform effects. Accordingly, these cohort-specific results are presented descriptively as performance observed within the respective cohort–platform configurations and are not interpreted as evidence of platform-independent performance.

Overall, the integrated targeted VOC panel-based classification framework showed stable discrimination performance and maintained classification performance between cancer and non-cancer samples within both cohorts, although cohort-specific differences in threshold-dependent metrics were observed.

## 3. Discussion

This study integrated canine cohorts recruited in Korea and Thailand and evaluated an HS-GC/MS-based targeted blood VOC panel within a single classification framework for discriminating cancer from non-cancer samples. The main significance of this study lies in evaluating the applicability of a common framework while incorporating cohorts from different countries into a single analytical system, without developing separate country-specific models optimized for individual countries. In the 30 repeated validations, AUROC and AUPRC were 0.926 ± 0.019 and 0.894 ± 0.023, respectively, and balanced accuracy, F1-score, MCC, and Cohen’s κ were also maintained relatively consistently across the repeated evaluations. These results indicate that the multivariate signal derived from the predefined targeted VOC panel provides meaningful information for classifying cancer and non-cancer samples and that this performance was repeatedly observed in a cohort-aware internal repeated-split validation setting within the integrated retrospective cohort rather than being limited to a single data split.

### 3.1. Integrated Multinational Framework for Canine Cancer Screening

Integrating multinational cohorts into a single framework for canine cancer screening has implications beyond simply increasing sample size. Differences across countries and institutions may involve breed composition, living environment, diet, clinical practice settings, cancer-type distribution, and disease stage, all of which can influence both VOC signals and classification performance. Therefore, international applicability cannot be adequately assessed based only on performance derived from a single country or a single cohort. In this study, the Korea and Thailand cohorts were not separated into distinct models. Instead, class-stratified partitioning was performed within each cohort, and the study was designed so that samples from both countries were included in both the training and validation sets. This cohort-aware integration represents a practical approach for evaluating the stability of a common classification framework across heterogeneous cohorts within an integrated retrospective dataset, without constructing separate country-specific models, rather than providing independent external validation.

In the repeated validation results, the seed-level ranges of AUROC and AUPRC were 0.890–0.958 and 0.852–0.940, respectively. These ranges indicate that performance variability existed across repeats, while also showing that discrimination performance was generally maintained at a high level across different random splits. In prediction model studies, performance based on a single training–validation split may be overestimated or underestimated depending on the specific partitioning structure. Therefore, it is important to report both the average performance level and its variability through repeated internal validation [21]. The mean, SD, 95% CI, and min–max range reported in this study quantitatively present this variability and support that the performance of the integrated framework was not dependent on a single seed.

The consistently high AUPRC observed in the validation setting with class imbalance is also important. AUROC is useful for evaluating overall ranking performance between cancer and non-cancer samples; however, when the positive class is the minority class, it may not fully reflect the practical utility of positive classification. In contrast, the PR curve directly reflects the precision–recall trade-off for the cancer class and therefore serves as an important complementary metric in imbalanced screening datasets [22]. In this study, the mean AUPRC was 0.894, substantially higher than the cancer prevalence baseline of 0.281 in the validation set. This indicates that the predicted cancer probability prioritized cancer samples into higher-score regions beyond what would be expected from class prevalence alone, suggesting that the framework also demonstrated meaningful performance in terms of positive-class enrichment.

The validation design of this study included procedures intended to improve the reliability of model evaluation. Class imbalance correction was applied only to the training data, and no resampling was applied to the validation data. Hyperparameter search was also performed within the training data, and the integrated validation set was not used during model optimization. This design preserved the original class distribution of the validation set and reduced the possibility of data leakage arising from resampling or tuning procedures. Therefore, the performance observed in this study can be interpreted as performance obtained on preserved internal validation subsets that were not used for resampling or hyperparameter optimization within each split, rather than as evidence of independent external validation.

### 3.2. Probability-Based Interpretation of Classification Scores

The classification output of this study should be interpreted not only as binary classification at a fixed threshold but also as the continuous distribution of predicted cancer probability. In the sample-level prediction score distribution, the median predicted cancer probability was 0.954 for cancer samples and 0.041 for non-cancer samples. This indicates clear score-level separation between cancer and non-cancer samples. These findings suggest that the multivariate signal derived from the targeted VOC panel contributes to classification not merely as changes in individual markers, but as a cancer-associated probability distribution.

At the same time, score overlap was also observed. Based on the sample-level mean probability, some cancer samples were located below the 0.5 threshold, whereas some non-cancer samples were located at or above the threshold. This finding supports the interpretation of the framework as a probability-based screening/classification approach rather than a definitive diagnostic tool. Low scores within the cancer group may be associated with various factors, including cancer type, disease stage, tumor burden, tumor metabolic activity, systemic inflammatory status, pretreatment clinical condition, or pre-analytical variability. Conversely, high scores within the non-cancer group may arise from non-neoplastic inflammation, infection, metabolic disease, environmental exposure, diet, medication, or other physiological variation. Such signal overlap and nonspecific variability have been recognized as important interpretive considerations in VOC-based biomarker research [23].

Among threshold-dependent metrics, specificity was 0.943 ± 0.022, which was higher than sensitivity at 0.772 ± 0.048. This indicates that, at the threshold of 0.5, the framework showed relatively stronger performance in assigning non-cancer samples to lower cancer probability regions. High specificity may be advantageous for reducing false-positive classifications and limiting the burden of unnecessary follow-up testing. However, the lower sensitivity relative to specificity indicates that some cancer samples may be located in lower-score regions; therefore, false-negative risk should also be considered in screening-oriented applications.

Therefore, the threshold of 0.5 should be understood as a reference point for comparing performance in the repeated validation setting of this study. In actual clinical application, the threshold may vary depending on the intended use. For early screening or high-risk group screening, threshold adjustment toward higher sensitivity may be necessary, whereas in settings where the burden of follow-up diagnostic testing is substantial, threshold selection considering specificity may be more important. In addition, because PPV and NPV are influenced by cancer prevalence in the validation set, predictive values in real-world clinical populations may vary according to cancer prevalence, age, breed, symptom status, and reason for hospital visit. Considering these factors, the score generated by this framework may have clearer clinical utility when presented as a cancer-associated probability or risk-oriented classification signal rather than as a simple positive/negative result.

Accordingly, the threshold-dependent results reported in this study should be regarded as performance estimates within the present retrospective validation dataset rather than as clinically validated operating characteristics of the CancerVET® system. In particular, the sensitivity, specificity, PPV, and NPV reported here should not be interpreted as fixed clinical performance estimates for routine veterinary screening populations because the underlying disease prevalence and clinical spectrum may differ from those represented in this dataset.

### 3.3. Cohort Heterogeneity and Framework Applicability

The cohort-specific analyses of the Korea and Thailand cohorts provided information on the behavior of the integrated classification framework within the two retrospective cohort–platform configurations examined in this study. AUROC and AUPRC remained at favorable levels in both cohorts, indicating that ranking-based discrimination between cancer and non-cancer samples was observed within each cohort. However, differences were observed in threshold-dependent metrics. In the Korea cohort, discrimination performance was maintained, with an AUROC of 0.882 ± 0.025 and an AUPRC of 0.822 ± 0.038, although sensitivity and F1-score were lower than those in the Thailand cohort. In contrast, the Thailand cohort showed stronger classification performance, with an AUROC of 0.960 ± 0.019, an AUPRC of 0.951 ± 0.021, sensitivity of 0.878 ± 0.046, and an F1-score of 0.905 ± 0.039.

These differences are consistent with the sample-level score distributions. Cancer samples in the Korea cohort were more broadly distributed toward lower-probability regions than those in the Thailand cohort, resulting in lower sensitivity and F1-score at the fixed threshold of 0.5. This finding does not indicate that ranking information between cancer and non-cancer samples was lost in the Korea cohort. Rather, it shows that the location and spread of the score distribution differed from those in the Thailand cohort. Thus, the lower threshold-dependent metrics despite maintained AUROC and AUPRC demonstrate that ranking ability and fixed-threshold operating characteristics provide different information.

Crucially, it should be noted that the national cohorts were completely confounded with the analytical instrument platforms in this study (Korea with Agilent Technologies (Santa Clara, CA, USA) and Thailand with Thermo Fisher Scientific (Waltham, MA, USA)). Although the classification signal was maintained in both cohorts, with AUROC values exceeding 0.88, the present analysis cannot determine whether the observed cohort-specific differences or the preservation of discrimination performance are attributable to biological and clinical characteristics of the cohorts, analytical platform differences, or their combined effects. Therefore, the present findings should not be interpreted as demonstrating platform-independent robustness of the targeted VOC panel. Rather, they demonstrate that the integrated classification framework retained a classification signal under the specific combination of cohorts and analytical platforms examined in this retrospective dataset.

Cohort-specific differences should not be interpreted as indicating the superiority or inferiority of a particular national cohort. This study was not designed to develop country-specific models or to compare models between countries, but to evaluate cohort-level behavior when a single integrated framework was applied to the Korea and Thailand cohorts. Differences between countries may reflect the combined effects of various biological, clinical, and analytical factors, including breed composition, cancer-type distribution, disease stage, control group composition, clinical environment, sample handling conditions, and analytical batch. In particular, VOC signals can be influenced not only by tumor-associated metabolic changes but also by inflammation, oxidative stress, microbiome, diet, environmental exposure, and sample handling conditions. Therefore, performance differences between cohorts are unlikely to be explained by a single factor.

The analysis of age and sex/neuter status further supports the presence of cohort heterogeneity in this study population. Cancer samples were older than non-cancer samples, and the distribution of sex/neuter status differed across class and national cohort strata, particularly with respect to the proportion of neutered or spayed dogs. These differences are relevant for interpreting the cohort-specific score distributions and threshold-dependent metrics, because age and neuter status may be associated with clinical background, health-screening behavior, tumor prevalence, or other unmeasured biological and environmental factors. However, the present analysis was descriptive and does not establish whether age or sex/neuter status contributed directly to the observed classification scores or cohort-specific performance differences. The analysis of age and sex/neuter status further supports the presence of cohort heterogeneity in this study population. Cancer samples were older than non-cancer samples, and the distribution of sex/neuter status differed across class and national cohort strata, particularly with respect to the proportion of neutered or spayed dogs. These differences are relevant when interpreting cohort-specific score distributions and threshold-dependent metrics because age and sex/neuter status may be associated with clinical background, health-screening behavior, tumor prevalence, or other unmeasured biological and environmental factors. To further evaluate whether the marked age difference between the cancer and non-cancer groups could substantially account for the observed classification performance, we performed a sensitivity analysis restricted to dogs aged ≥8 years. The age-restricted analysis included 336 dogs and yielded a mean AUROC of 0.911 ± 0.029 and a mean AUPRC of 0.909 ± 0.027, with sensitivity of 0.763 ± 0.055 and specificity of 0.925 ± 0.038 at the predefined threshold of 0.5. These findings provide supportive evidence that the observed classification signal was not solely attributable to the age difference between the cancer and non-cancer groups. However, the age-restricted cohort was smaller than the primary cohort, and other potential biological, clinical, and analytical sources of heterogeneity remained. Therefore, this sensitivity analysis does not establish the independent contribution of age or exclude residual confounding and should be interpreted as supportive rather than definitive evidence of age-independent classification performance.

These findings support a balanced interpretation of the integrated framework. The framework retained a classification signal between cancer and non-cancer samples in both national cohorts and demonstrated stable discrimination performance across repeated internal validations within the integrated retrospective cohort. At the same time, differences in cohort-specific threshold-dependent metrics indicate that operating characteristics may vary across cohorts. Because cohort and analytical platform were completely confounded in the present dataset, these differences cannot be attributed independently to either factor. Therefore, the present study provides evidence of classification performance under the specific multinational cohort and analytical-platform configuration examined here, but does not establish platform-independent generalizability. Further validation using newly recruited cohorts and analytical settings in which cohort and platform effects can be evaluated separately will be necessary.

### 3.4. Analytical and Clinical Implications of a Targeted Blood VOC Panel

The targeted VOC panel approach used in this study has practical advantages that distinguish it from conventional untargeted volatilomics. Although untargeted approaches are useful in the biomarker discovery stage, issues related to compound identification, peak annotation, analytical reproducibility, platform transferability, and environmental contamination become important when clinical application is considered. In contrast, a targeted panel may be more practical for standardizing analytical procedures and translating them into clinical workflows, because predefined compounds are measured repeatedly. In this study, nine markers consisting of aromatic compounds and straight-chain alkanes were analyzed, and panel-level multivariate classification performance was evaluated rather than the directional behavior of individual VOCs.

This approach provides an important interpretive balance in VOC-based cancer screening. Straight-chain alkanes may be associated with lipid peroxidation and oxidative stress, whereas aromatic compounds may be related to tumor-associated metabolic changes or altered metabolic clearance processes. However, this does not imply that these compounds are tumor-specific markers when considered individually. The exclusion of aldehyde markers from the targeted panel in this study was related to a strategy for reducing nonspecific VOC variation that may occur under inflammatory, infectious, or non-neoplastic oxidative stress conditions. Nevertheless, the use of a targeted panel does not eliminate all biological or analytical confounding. Therefore, the findings of this study should be interpreted as the performance of the multivariate classification signal generated by the targeted panel as a whole, rather than as evidence of tumor specificity for individual VOCs. Accordingly, biological plausibility was used as a rationale for panel selection, but was not considered evidence of cancer specificity, diagnostic specificity, or causal involvement in tumor development for any individual VOC.

HS-GC/MS analysis using whole blood also has relevance for clinical translation. Compared with breath-based analysis, blood-based VOC analysis may be affected differently by sampling behavior, respiratory patterns, and oral or respiratory tract conditions, and it has the advantage of assessing metabolic and physiological changes reflected in systemic circulation. In this study, a simplified preparation procedure was used in which whole blood was directly applied to a headspace vial without an additional separation step. This approach may offer advantages in terms of sample-processing simplicity, repeatability, centralized laboratory operation, and workflow standardization required in real-world clinical settings.

At the same time, blood VOC analysis is an area in which pre-analytical standardization is critical. Anticoagulants, blood collection procedures, storage temperature, freeze–thaw events, transportation time, headspace incubation conditions, matrix effects, and instrument conditions can all influence VOC signals. In this study, procedures intended to reduce pre-analytical variability were applied, including the use of citrate tubes, powder-free gloves, frozen transportation, sample verification at the central analytical laboratory, and centralized sample management. Although the same sample preparation procedure was applied across the analytical platforms, the Korea cohort was analyzed exclusively using the Agilent platform and the Thailand cohort exclusively using the Thermo Fisher platform. Because cohort and analytical platform were completely confounded, the present study cannot determine the independent contribution of analytical platform to the observed cohort-specific performance. Therefore, the use of two analytical platforms in this retrospective dataset should be interpreted as part of the specific cohort–platform configuration examined rather than as evidence of platform-independent robustness. Evaluation across analytical platforms in future studies using a design that allows cohort and platform effects to be disentangled will be necessary to assess analytical transportability.

### 3.5. Limitations and Future Directions

A strength of this study is that it evaluated an integrated framework without constructing separate country-specific models, using repeated internal validation that incorporated multinational cohorts. However, because the training and validation subsets were repeatedly generated from the same integrated retrospective cohort, these results should not be interpreted as independent external validation. Rather, the findings demonstrate performance stability under internal repeated-split validation within the integrated cohort. Accordingly, the present study does not establish clinical applicability or external generalizability. It should therefore not be considered a clinical validation study of the CancerVET® system. Future studies should evaluate transportability and external generalizability in newly recruited populations, institutions, countries, and clinical settings through independent prospective validation using a locked analytical pipeline.

Second, the causes of cohort-specific differences cannot be disentangled from the current analysis alone. Although the metadata analysis showed differences in age distribution and sex/neuter status across class and cohort strata, these variables were summarized for cohort characterization and were not incorporated into covariate-adjusted performance analyses. Therefore, it remains unclear whether the wider and lower-shifted cancer score distribution in the Korea cohort was driven by age, sex/neuter status, cancer-type composition, disease stage, tumor burden, breed, body weight, control group composition, inflammatory comorbidities, medication, diet, environmental exposure, sample handling conditions, or analytical batch. Specifically, the complete confounding between country cohort and analytical platform (Korea/Agilent vs. Thailand/Thermo) means that cohort-specific performance differences cannot be entirely disentangled from instrument-related analytical variability. Future studies should include subgroup analyses incorporating age group, sex/neuter status, breed, body weight, cancer type, disease stage, inflammatory comorbidity, and other clinical covariates. Such analyses would help interpret cohort differences not as a simple effect of geography, but as the combined result of biological, clinical, individual-level, and operational factors.

Third, this study evaluated binary classification between cancer and non-cancer samples and did not assess cancer-type-specific or organ-specific classification performance. The tumour-type distribution provided in the Supplementary Information is intended to characterize the composition of the cancer cohort and should not be interpreted as evidence of tumour-type-specific classification performance. A pan-cancer screening approach has the advantage of evaluating multiple cancer types within a single framework, but it also entails heterogeneity within the cancer group. In addition, because the non-cancer group in this study consisted of controls without tumor-associated abnormalities, the ability to discriminate cancer from clinical controls with benign tumors, inflammatory diseases, infectious diseases, metabolic diseases, or geriatric comorbidities should be evaluated separately. Such disease-control validation is important for understanding real-world clinical specificity and mechanisms underlying false-positive classifications.

Finally, calibration of the predicted cancer probability and threshold selection are important areas for future clinical implementation. Although threshold-dependent metrics were calculated using a threshold of 0.5 in this study, the optimal threshold in real-world screening workflows may vary depending on the target population, disease prevalence, and burden of subsequent confirmatory testing. Additional calibration curve analysis, Brier score assessment, decision curve analysis, and threshold sensitivity analysis would help clarify how the framework score should be interpreted and used clinically.

A further limitation concerns reproducibility. The reproducibility of the validation and performance-evaluation framework should be distinguished from reproducibility of the proprietary CancerVET® classification algorithm itself. The procedural aspects of the validation framework are described in the manuscript, including the cohort-aware training–validation split, training-only application of SMOTE, restriction of hyperparameter optimization to the training data, five-fold cross-validation within the training data, use of the selected hyperparameter configuration across repeated evaluations, predefined thresholding, and repetition across 30 random seeds. These specifications allow the methodological design of the validation procedure to be independently assessed. However, the exact data transformations, input-variable construction, and internal implementation of the proprietary classification algorithm cannot be independently reproduced from the information disclosed in this manuscript. Therefore, the repeated analyses demonstrate stability of the reported performance under the specified validation framework and should not be interpreted as independent reproduction of the proprietary algorithm itself.

## 4. Materials and Methods

### 4.1. Study Population and Case Classification

This study was conducted in dogs that presented to participating veterinary hospitals in Korea and Thailand. In Thailand, samples were collected under the supervision of Kasetsart University Veterinary Teaching Hospital, Bangkhen Campus, in collaboration with the Oncology Clinic, Small Animal Teaching Hospital, Faculty of Veterinary Science, Chulalongkorn University, and private veterinary hospitals in the Bangkok metropolitan area. In Korea, samples were collected through a network of multiple participating veterinary hospitals.

A total of 590 dogs were enrolled in this retrospective study. The study population consisted of 167 dogs in the malignant tumor group and 423 dogs in the non-cancer control group. By country, 321 dogs were recruited in Korea and 269 dogs in Thailand (Table 2).

Dogs were included in the malignant tumor group if they had been confirmed to have malignant tumors by histopathological examination or diagnostic imaging. The non-cancer control group was limited to dogs aged ≥1 year with no history of tumors and no tumor-associated abnormalities identified during routine health examinations performed within the preceding 6 months. In both groups, dogs that had undergone surgery, chemotherapy, or radiation therapy within the preceding 3 months, as well as pregnant or lactating dogs, were excluded from the study.

### 4.2. Blood Sample Collection and Handling

**Figure 5:**
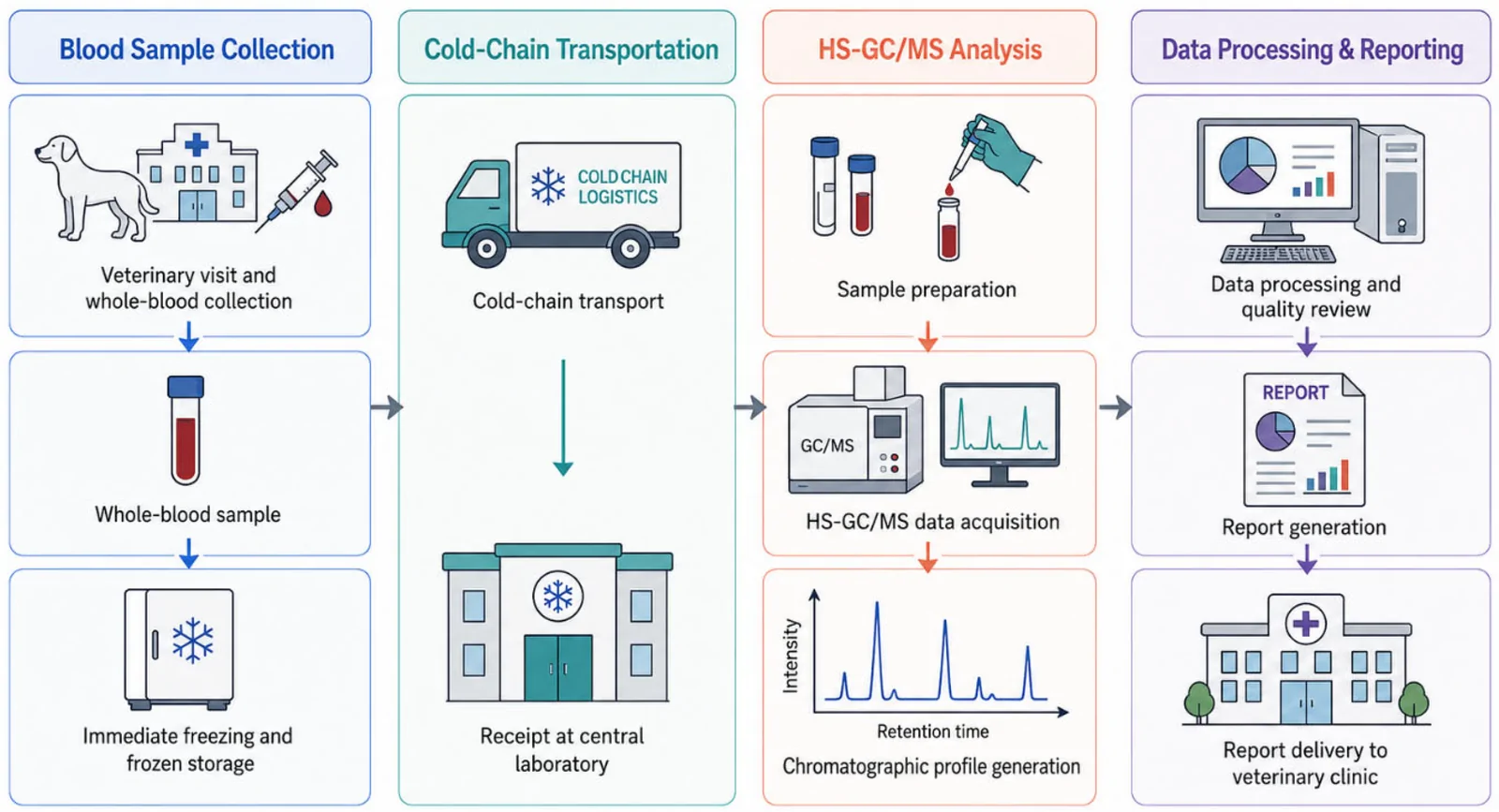
Schematic overview of the CancerVET^®^ platform workflow for canine cancer screening. Whole-blood samples were collected from dogs during veterinary visits, immediately frozen and stored under frozen conditions, and transported to a central laboratory through cold-chain logistics. HS-GC/MS-based blood VOC profiles were processed using the CancerVET^®^ data-processing and classification pipeline to generate screening reports for veterinary clinical use.

Blood samples were collected from the study subjects described in the Study Population and Case Classification section according to standardized sample collection and processing procedures uniformly applied at each participating institution (Figure 5). Before blood collection, breed, age, sex, neuter status, body weight, and relevant clinical information for each dog were recorded in an electronic case report form (eCRF) shared across institutions.

Whole-blood samples were collected by standard venipuncture using evacuated blood collection tubes containing 0.109 M sodium citrate as an anticoagulant (9NC citrate tube). To minimize contamination by exogenous VOCs, disposable powder-free gloves were worn throughout the blood collection procedure. Immediately after collection, the tubes were gently inverted to ensure adequate mixing of blood and anticoagulant. Approximately 1.5 mL of whole blood was targeted per dog, and the minimum acceptable volume was set at 1.2 mL to ensure that 1 mL of sample required for analysis could be secured.

All collected samples were assigned a unique identification number immediately after blood collection. Until transportation, samples were stored at ≤−4 °C, and when long-term storage was required, they were transferred to frozen storage conditions at ≤−40 °C. During all storage and handling procedures other than transportation, thawing and refreezing were strictly restricted, and exposure to room temperature was minimized. All samples were transported to the central analytical laboratory, MetaDx Inc. (Seoul, Republic of Korea), while maintaining frozen conditions using cooling materials such as dry ice and ice packs. Upon arrival, sample identification information, container condition, and storage condition were verified. All samples were managed at a single analytical laboratory according to the same sample handling procedures and quality control system and were subsequently used for HS-GC/MS-based VOC analysis.

### 4.3. Sample Preparation and HS-GC/MS Analysis

The sample preparation procedure was designed in a simplified format to support applicability in routine clinical settings. For this purpose, whole blood without an additional separation step was selected as the analytical specimen, and a headspace-based analytical system capable of directly separating volatile organic compounds from the sample was adopted. This design was intended to minimize operator dependency and processing time during sample handling.

Specifically, 1 mL of whole blood was aliquoted into a 20 mL headspace vial, followed by the addition of 12 mL of saturated NaCl solution adjusted to a urea concentration of 2.5 M. The prepared vial was incubated at 99 °C for 40 min and then introduced into the HS-GC/MS system for analysis.

Two HS-GC/MS platforms from different manufacturers were operated for the analysis of samples collected in Korea and Thailand. The first platform was a Thermo Fisher Scientific system (Waltham, MA, USA) consisting of a Trace 1610 gas chromatograph, a Triplus 500 headspace autosampler, and an ISQ 7610 single quadrupole mass spectrometer. The second platform was an Agilent Technologies system (Santa Clara, CA, USA) consisting of an 8890 gas chromatograph, a 5977C GC/MSD, and a PAL RSI 85 (Series 2) autosampler. In this retrospective setting, the Korea cohort was entirely analyzed using the Agilent platform, while the Thailand cohort was analyzed using the Thermo Fisher platform. The same sample preparation procedure, including incubation conditions, was applied on both platforms.

Separation of the target analytes in the samples was performed on both platforms using the same TG-624SilMS capillary column (0.32 mm i.d. × 30 m, 0.18 µm film thickness) and a stepwise thermal programming protocol over 30 min. The temperature program consisted of three steps: an initial hold at 50 °C for 5 min followed by heating to 100 °C at 10 °C/min, heating from 100 °C to 120 °C at 20 °C/min, and heating from 120 °C to 260 °C at 30 °C/min followed by a 4.33 min hold. Detailed instrumental parameters for the two platforms are provided in Supplementary Table S1.

### 4.4. Targeted Biomarker Panel

In this study, we analyzed nine targeted biomarkers selected a priori on the basis of biological rationale and analytical suitability, rather than using an untargeted analytical approach covering the entire volatile organic compound profile. The targeted panel consisted of five aromatic compounds and four straight-chain alkanes. The five aromatic compounds included benzene, toluene, ethylbenzene, xylene, and styrene, and the four straight-chain alkanes were undecane (C11), dodecane (C12), tridecane (C13), and tetradecane (C14). The selection of the C11–C14 alkane range was based on a balance between biological relevance and headspace analytical suitability. Lower-molecular-weight *n*-alkanes are highly volatile and may show limited retention in biological matrices, whereas higher-molecular-weight *n*-alkanes have lower vapor pressures and consequently reduced transfer into the headspace under the incubation conditions used in this study. The C11–C14 range therefore provided a practical intermediate range for reproducible headspace analysis while remaining biologically relevant to oxidative lipid metabolism and alkane generation. Previous studies have reported straight-chain alkanes across a broad carbon-number range in cancer-related VOC profiles and have linked alkane production to reactive oxygen species-mediated lipid peroxidation and subsequent oxidative metabolism [24,25].

The aromatic compounds were selected for a complementary biological and analytical rationale. Benzene, toluene, ethylbenzene, xylene, and styrene belong to a closely related group of volatile aromatic hydrocarbons that have repeatedly been detected in human breath, blood, or tissue VOC studies and have been investigated in relation to cancer-associated VOC patterns, particularly in studies of lung and other solid tumors [21,22,23]. In lung cancer studies, several of these compounds, including benzene, toluene, ethylbenzene, styrene, and xylenes, have been reported among VOCs that differed between cancer patients and control groups or were detected in both cancerous tissues and exhaled breath [21,22]. Their inclusion in the present panel was therefore intended to capture a complementary aromatic VOC component of cancer-associated metabolic and physiological alterations, rather than to assume that any single aromatic compound is tumor-specific. Importantly, the biological origin of these compounds may be heterogeneous, because aromatic VOC concentrations can be influenced by endogenous metabolism as well as environmental or occupational exposure, smoking, and other exogenous sources [21]. Accordingly, the five aromatic compounds were treated as components of a multivariate panel rather than as individually validated cancer biomarkers.

For the straight-chain alkanes, the biological rationale was based primarily on oxidative lipid metabolism. Increased reactive oxygen species can promote lipid peroxidation of polyunsaturated fatty acids in biological membranes, generating volatile hydrocarbons that may enter the circulating or exhaled VOC profile [24,25]. In addition, cytochrome P450-dependent oxidation contributes to the metabolism and elimination of volatile alkanes, providing a biological basis for considering these compounds as part of an oxidative-stress-related VOC profile [24]. However, straight-chain alkanes are not specific to malignant disease, because lipid peroxidation and oxidative stress can also occur in non-neoplastic inflammatory and metabolic conditions [25]. For this reason, their inclusion in the present study was intended to capture a biologically relevant component of the overall VOC profile rather than to establish individual alkane-specific cancer biomarkers. In addition, aldehyde compounds, including hexanal, are known to vary not only in association with tumor-specific changes but also under inflammatory, infectious, and other oxidative stress-related conditions [26]. Therefore, these compounds were excluded from the analytical targets to minimize confounding by nonspecific physiological or pathological factors. This strategy enabled the construction of a minimal targeted panel with clinical interpretability and analytical reproducibility, in contrast to conventional approaches that use the entire VOC spectrum.

All biomarkers, including the representative VOCs included in CancerVET®, were subjected to peak integration using TraceFinder 5.1 software (Thermo Fisher Scientific, Waltham, MA, USA) and MassHunter Quant software (Agilent Technologies, Santa Clara, CA, USA) before data analysis.

### 4.5. Model Development and Validation Strategy

The targeted VOC panel-based classification pipeline evaluated in this study includes proprietary data-processing procedures and machine learning-based classification steps. Detailed data transformation methods, input variable construction, and the internal implementation structure of the classification algorithm were not disclosed because they constitute proprietary technology. In contrast, the procedural components of the validation and performance-evaluation framework are explicitly described in this manuscript, including cohort-aware data partitioning, the validation-set proportion, application of class-imbalance correction only to the training data, restriction of hyperparameter optimization to the training data, the cross-validation strategy used for optimization, the use of a fixed hyperparameter configuration across repeated evaluations, the predefined classification threshold, performance metrics, and the number of repeated random seeds. These details allow independent assessment of the validation and performance-evaluation procedures, although the exact internal implementation of the proprietary CancerVET® classification algorithm cannot be independently reproduced from the information disclosed in this manuscript.

**Figure 6:**
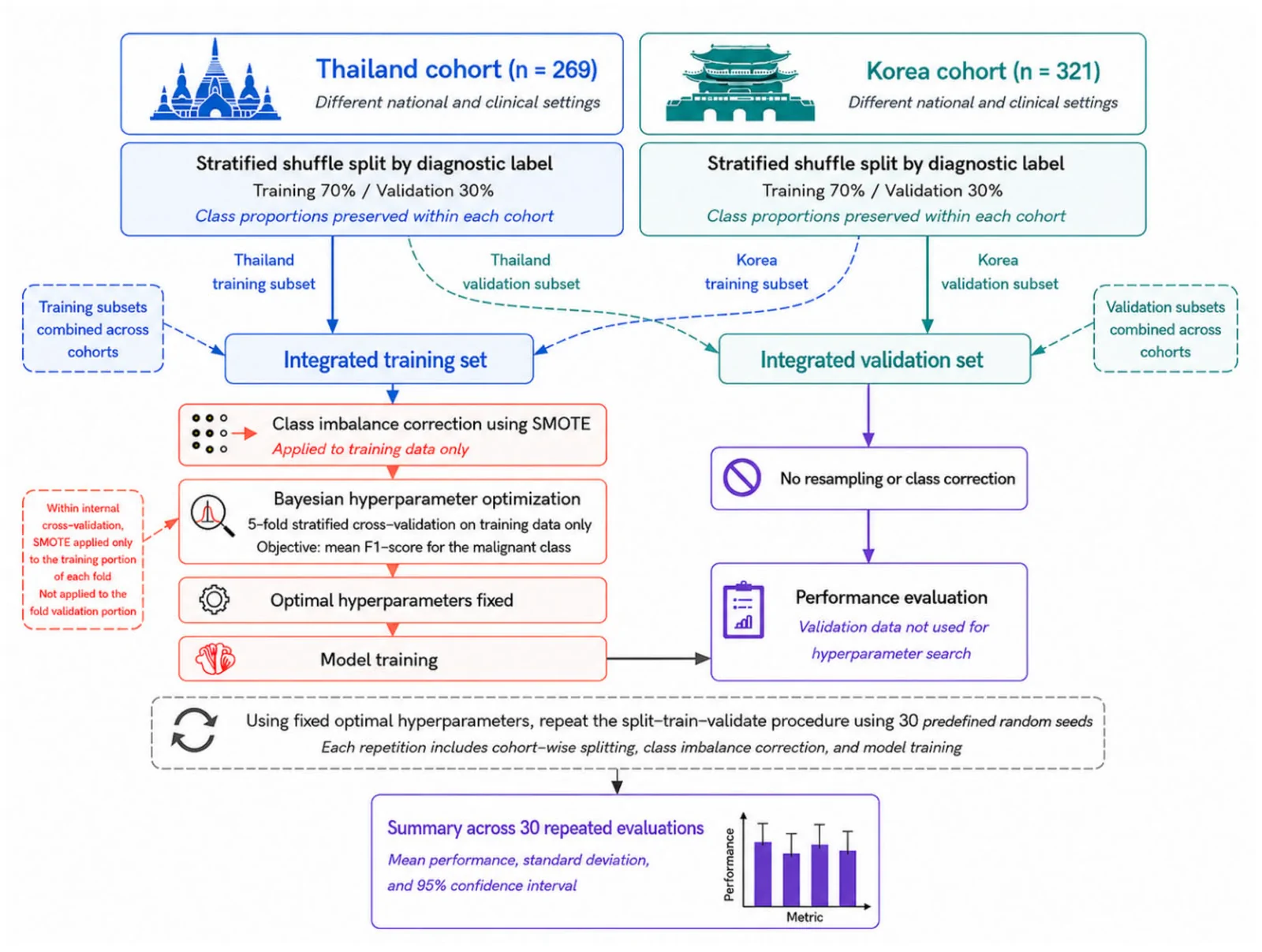
Workflow of model development and internal repeated-split validation strategy within the integrated retrospective cohort. The Thailand and Korea cohorts were each divided into training and validation subsets using stratified shuffle splitting based on diagnostic labels. The training subsets generated from each cohort were combined to form the integrated training set, and the validation subsets were combined to form the integrated validation set. Class imbalance correction, hyperparameter optimization, and model training were performed exclusively using the training data, whereas no resampling or class-balancing procedure was applied to the validation data. The entire training–validation procedure was repeated across 30 random seeds to assess the average performance and variability of the integrated classification framework within the integrated retrospective cohort. Because both the training and validation subsets were generated from the same integrated retrospective cohort, this procedure represents internal repeated-split validation rather than independent external validation. Model hyperparameters were optimized using Bayesian optimization. Hyperparameter search was restricted to the training data of the initial development split, and the mean F1-score for the cancer class obtained from 5-fold stratified cross-validation within the training data was used as the objective function. The integrated validation set was not used during hyperparameter search. The hyperparameter configuration identified through this search was applied consistently in the subsequent repeated evaluations, ensuring that performance differences across repeated validations were not influenced by hyperparameter re-optimization.

The analytical dataset in this study consisted of the Thailand cohort (n = 269) and the Korea cohort (n = 321). The two cohorts were collected in different countries and clinical settings, and these differences may influence data distributions across sample collection, storage, transportation, and analytical preprocessing procedures. Therefore, the data partitioning procedure was designed to maintain the representativeness of each cohort in both the training and validation stages. Rather than simply pooling the entire dataset and performing random partitioning, stratified shuffle splitting based on class labels was performed separately within the Thailand and Korea cohorts, with a test size of 30%. The training subsets generated from each cohort were then combined to form the integrated training set, and the validation subsets generated from each cohort were combined to form the integrated validation set (Figure 6). Stratification was performed to preserve, as much as possible, the proportions of cancer and non-cancer samples within each cohort in both the training and validation sets. This procedure ensured that samples from both the Korea and Thailand cohorts were included in both the training and validation sets and prevented either cohort from being disproportionately assigned to one dataset. Because both the training and validation subsets were generated from the same integrated retrospective cohort, this design represents internal validation rather than independent external validation.

Synthetic Minority Over-sampling Technique (SMOTE) was applied to correct class imbalance between cancer and non-cancer samples within the training set. Class imbalance correction was performed only on the training data, and no resampling or class-balancing procedure was applied to the validation data. In addition, when internal cross-validation was performed during model optimization, SMOTE was applied only to the training portion of each fold and not to the validation portion of that fold. This approach preserved the original class distribution of the validation data and minimized the possibility of data leakage due to resampling.

To assess performance variability associated with data partitioning, the same cohort-aware stratified split procedure was repeated using 30 different random seeds within the integrated retrospective cohort. In each repeat, cohort-specific training–validation splitting, class imbalance correction within the training set, and model training were performed independently according to the corresponding split. This internal repeated-split validation allowed evaluation of both the average level and variability of classification performance associated with data partitioning, rather than relying on a single training–validation split.

All major performance results were summarized based on seed-level predictions generated from the 30 repeated internal validation runs. Results from a single split were not presented as representative performance. Overall performance was reported as the mean, standard deviation, 95 % confidence interval, and min–max range of the metrics obtained from the 30 seeds. Cohort-specific performance was calculated in the same manner after stratifying the validation predictions from each repeated internal validation run by the Korea and Thailand cohorts. This approach enabled assessment of not only overall performance in the integrated retrospective cohort under repeated internal validation but also the maintenance of performance within each national cohort. To avoid performance estimation based on a single random seed, internal repeated validation was performed using 30 predefined random seeds, thereby evaluating performance variability associated with data partitioning and supporting reproducibility of the validation framework.

### 4.6. Statistical Analysis and Performance Metrics

Age and sex/neuter status metadata were summarized to characterize the study population. Age was reported as the mean ± SD, median, interquartile range (IQR), and range. Biological sex and sex/neuter status were summarized as counts and percentages. Exploratory comparisons of age distribution between cancer and non-cancer samples and between the Korea and Thailand cohorts were performed using the Mann–Whitney U test. Categorical distributions of biological sex and sex/neuter status were compared using the chi-square test. These baseline characteristic analyses were performed for cohort characterization only and were not used as model inputs, data-partitioning stratification factors, or covariate-adjustment procedures for performance estimation. As a sensitivity analysis for potential age-related confounding, an additional analysis was performed after restricting the study population to dogs aged ≥8 years. The same repeated internal validation framework described for the primary analysis was applied to the age-restricted cohort. The cohort-aware training–validation splitting strategy, class-imbalance correction restricted to the training data, hyperparameter configuration, classification threshold, and repeated evaluation procedure were maintained as described above. AUROC, AUPRC, sensitivity, and specificity were evaluated using the same procedures as in the primary analysis. Because the age-restricted analysis involved a smaller sample size, it was considered a supportive sensitivity analysis rather than a definitive assessment of the independent contribution of age to classification performance.

Statistical analyses were performed to evaluate the repeated internal validation performance of the integrated targeted VOC panel-based classification framework. Cancer samples were defined as the positive class, and non-cancer samples as the negative class. Class assignment was performed using a predicted cancer probability threshold of 0.5. Samples with a predicted cancer probability ≥ 0.5 were classified as cancer, whereas those with a predicted cancer probability < 0.5 were classified as non-cancer. In each validation run, threshold-dependent performance metrics were calculated based on agreement between the predicted class and the reference class label.

Threshold-dependent performance was assessed using accuracy, balanced accuracy, sensitivity, specificity, precision/positive predictive value (PPV), negative predictive value (NPV), F1-score, false-negative rate (FNR), false-positive rate (FPR), Matthews correlation coefficient (MCC), and Cohen’s κ. Accuracy was calculated as the proportion of correctly classified samples among all samples. Sensitivity was defined as the proportion of cancer samples correctly classified as cancer, and specificity as the proportion of non-cancer samples correctly classified as non-cancer. Balanced accuracy was calculated as the average of sensitivity and specificity. PPV was calculated as the proportion of true cancer samples among samples classified as cancer, and NPV as the proportion of true non-cancer samples among samples classified as non-cancer. The F1-score was calculated as the harmonic mean of precision and sensitivity. FNR was defined as the proportion of cancer samples classified as non-cancer, and FPR as the proportion of non-cancer samples classified as cancer. MCC and Cohen’s κ were included as complementary metrics accounting for class imbalance and chance agreement.

Threshold-independent discrimination performance was evaluated using the area under the receiver operating characteristic curve (AUROC) and the area under the precision–recall curve (AUPRC). AUROC was calculated from predicted cancer probabilities and binary reference labels. AUPRC was calculated as average precision, with cancer defined as the positive class. Because class imbalance was present in the validation set, precision–recall curve analysis was performed in addition to ROC curve analysis to evaluate positive-class enrichment for the cancer class relative to the cancer prevalence baseline.

Performance metrics were calculated independently for each of the 30 repeated internal validation runs. Overall performance was summarized using the mean, sample standard deviation (SD), 95 % confidence interval (CI), and min–max range of the seed-level metric values. The 95 % CI for the mean was calculated using a two-sided t-distribution based on the 30 seed-level metric values. To evaluate cohort-specific performance, validation predictions from each seed were stratified by the Korea and Thailand cohorts, and the same metrics were calculated separately for each cohort. Cohort-specific metrics were also summarized as the mean, SD, 95 % CI, and min–max range across the 30 repeated internal validation runs. Performance comparisons between cohorts were conducted descriptively within the integrated validation framework and were not interpreted as independent inferential comparisons of country-specific model superiority.

For ROC curve visualization, each seed-level ROC curve was interpolated onto a common false-positive rate grid, and the mean ROC curve and ±1 SD band were calculated across repeated validations. For the precision–recall curve, each seed-level PR curve was interpolated onto a common recall grid, and the mean PR curve and ±1 SD band were calculated. The random-classifier baseline in the PR curve was defined as the prevalence of cancer samples in the validation set.

Prediction score distribution was evaluated using predicted cancer probability. Because the same sample could be included in the validation set across multiple validation runs, predicted cancer probabilities across validation occurrences were averaged for each sample to calculate the sample-level mean predicted cancer probability. This value was used to compare score distributions between cancer and non-cancer samples and among cohort-specific class groups. Sample-level threshold counts were calculated based on the sample-level mean predicted cancer probability averaged across validation occurrences, rather than on confusion outcomes from individual repeated validation runs.

All statistical analyses and figure generation were performed in Python 3.13.5. Data processing and numerical calculations used pandas 2.2.3 and NumPy 2.3.5. Performance metrics, ROC curve analysis, and precision–recall curve analysis used scikit-learn 1.8.0. Statistical calculations, including 95% confidence intervals, used SciPy 1.17.0, and final figures were generated using Matplotlib 3.10.8.

## 5. Conclusions

This study evaluated an integrated classification framework using an HS-GC/MS-based targeted blood VOC panel for discriminating cancer from non-cancer samples in a retrospective cohort comprising dogs recruited in Korea and Thailand. Across 30 repeated cohort-aware training–validation splits, the framework demonstrated consistent discrimination performance within the integrated retrospective cohort, with high AUROC and AUPRC values and clear probability-based separation between cancer and non-cancer samples. Cohort-specific analyses showed that the classification signal was retained within both the Korea and Thailand cohort–platform configurations, although differences in threshold-dependent performance were observed. These findings demonstrate performance stability under repeated internal validation within the specific integrated retrospective dataset and should not be interpreted as independent external validation, clinical validation, or evidence of platform-independent generalizability. Because the Korea and Thailand cohorts were completely confounded with the Agilent and Thermo Fisher analytical platforms, respectively, the independent contributions of cohort characteristics and analytical platform cannot be determined from the present data. Independent prospective validation using newly recruited samples from separate institutions and clinical populations, together with clinically relevant disease-control evaluation, cohort-specific calibration, evaluation across analytical platforms in a design that permits cohort and platform effects to be disentangled, and assessment of clinical utility within real-world workflows, is required to determine the external generalizability and clinical applicability of the framework.

## Figures and Tables

**Table 1:** Repeated validation performance of the integrated classification framework.

Performance Metric	Mean ± SD	95% CI	Min–Max Range
AUROC	0.926 ± 0.019	0.919–0.933	0.890–0.958
AUPRC	0.894 ± 0.023	0.885–0.903	0.852–0.940
Balanced accuracy	0.858 ± 0.024	0.849–0.866	0.811–0.910
Accuracy	0.895 ± 0.018	0.888–0.902	0.860–0.933
Sensitivity	0.772 ± 0.048	0.754–0.790	0.680–0.860
Specificity	0.943 ± 0.022	0.935–0.951	0.906–0.992
Precision/PPV	0.845 ± 0.052	0.825–0.864	0.771–0.972
NPV	0.914 ± 0.016	0.908–0.920	0.886–0.946
F1-score	0.805 ± 0.033	0.793–0.818	0.737–0.878
MCC	0.736 ± 0.046	0.719–0.754	0.643–0.831
Cohen’s κ	0.734 ± 0.045	0.717–0.751	0.641–0.831

SD: standard deviation; CI: confidence interval; AUROC: area under the receiver operating characteristic curve; AUPRC: area under the precision–recall curve; PPV: positive predictive value; NPV: negative predictive value; MCC: Matthews correlation coefficient; Cohen’s κ: Cohen’s kappa coefficient.

**Table 2:** Cohort and class distribution for repeated internal validation.

Cohort	Dataset	Total, n	Non-Cancer, n (%)	Cancer, n (%)
Integrated cohort	Full cohort	590	423 (71.7)	167 (28.3)
	Training set per repeat	412	295 (71.6)	117 (28.4)
	Validation set per repeat	178	128 (71.9)	50 (28.1)
Korea	Full cohort	321	232 (72.3)	89 (27.7)
	Training set per repeat	224	162 (72.3)	62 (27.7)
	Validation set per repeat	97	70 (72.2)	27 (27.8)
Thailand	Full cohort	269	191 (71.0)	78 (29.0)
	Training set per repeat	188	133 (70.7)	55 (29.3)
	Validation set per repeat	81	58 (71.6)	23 (28.4)

## Data Availability

The raw data from this study are available upon reasonable request, with the consent of all authors.

## Supplementary Materials

The following supporting information can be downloaded at: https://www.mdpi.com/article/10.3390/ijms27188016/s1.

## Abbreviations

AUPRC	area under the precision–recall curve
AUROC	area under the receiver operating characteristic curve
CI	confidence interval
CYP	cytochrome P450
eCRF	electronic case report form
FNR	false-negative rate
FPR	false-positive rate
GC-MS	gas chromatography–mass spectrometry
GC/MSD	gas chromatography/mass selective detector
HS-GC/MS	headspace–gas chromatography/mass spectrometry
IACUC	Institutional Animal Care and Use Committee
IQR	interquartile range
MCC	Matthews correlation coefficient
NPV	negative predictive value
PPV	positive predictive value
PR	precision–recall
PSA	prostate-specific antigen
ROC	receiver operating characteristic
ROS	reactive oxygen species
SD	standard deviation
SMOTE	Synthetic Minority Over-sampling Technique
VOC	volatile organic compound

## References

[1] Schiffman J.D., Breen M. (2015). Comparative oncology: What dogs and other species can teach us about humans with cancer. Philos. Trans. R. Soc. Lond. B Biol. Sci..

[2] Lemon S., Zapka J., Puleo E., Luckmann R., Chasan-Taber L. (2001). Colorectal cancer screening participation: Comparisons with mammography and prostate-specific antigen screening. Am. J. Public Health.

[3] Barrett B., McKenna P. (2011). Communicating benefits and risks of screening for prostate, colon, and breast cancer. Fam. Med..

[4] Ulyte A., Wei W., Dressel H., Gruebner O., von Wyl V., Bähler C., Blozik E., Brüngger B., Schwenkglenks M. (2020). Variation of colorectal, breast and prostate cancer screening activity in Switzerland: Influence of insurance, policy and guidelines. PLoS ONE.

[5] Wright K., Shkabari B., Booth C., Knopf K., Tregear M., Gyawali B. (2025). Congruence of cancer screening recommendations between the USPSTF and the top ten US cancer centers: A cross sectional study. eClinicalMedicine.

[6] Flory A., Kruglyak K.M., Tynan J.A., McLennan L.M., Rafalko J.M., Fiaux P.C., Hernandez G.E., Marass F., Nakashe P., Ruiz-Perez C.A. (2022). Clinical validation of a next-generation sequencing-based multi-cancer early detection “liquid biopsy” blood test in over 1,000 dogs using an independent testing set: The CANcer Detection in Dogs (CANDiD) study. PLoS ONE.

[7] Flory A., McLennan L., Peet B., Kroll M., Stuart D., Brown D., Stuebner K., Phillips B., Coomber B.L., Woods J.P. (2023). Cancer detection in clinical practice and using blood-based liquid biopsy: A retrospective audit of over 350 dogs. J. Vet. Intern. Med..

[8] Vander Heiden M.G., Cantley L.C., Thompson C.B. (2009). Understanding the Warburg effect: The metabolic requirements of cell proliferation. Science.

[9] Woollam M., Siegel A.P., Munshi A., Liu S., Tholpady S., Gardner T., Li B.-Y., Yokota H., Agarwal M. (2023). Canine-Inspired Chemometric Analysis of Volatile Organic Compounds in Urine Headspace to Distinguish Prostate Cancer in Mice and Men. Cancers.

[10] Miekisch W., Schubert J.K., Noeldge-Schomburg G.F. (2004). Diagnostic potential of breath analysis—Focus on volatile organic compounds. Clin. Chim. Acta.

[11] Oyerinde A.S., Selvaraju V., Babu J.R., Geetha T. (2023). Potential Role of Oxidative Stress in the Production of Volatile Organic Compounds in Obesity. Antioxidants.

[12] Mochalski P., Leja M., Ślefarska-Wolak D., Mezmale L., Patsko V., Ager C., Królicka A., Mayhew C.A., Shani G., Haick H. (2023). Identification of Key Volatile Organic Compounds Released by Gastric Tissues as Potential Non-Invasive Biomarkers for Gastric Cancer. Diagnostics.

[13] Yu Y., Zhang N., Mai Y., Ren L., Chen Q., Cao Z., Chen Q., Liu Y., Hou W., Yang J. (2023). Correcting batch effects in large-scale multiomics studies using a reference-material-based ratio method. Genome Biol..

[14] Ahn K.-G., Choi R., Gwak S., Choi I., Jang G., Kim J.-W., Kim G.A. (2025). A Novel Sample Preparation Method for GC-MS Analysis of Volatile Organic Compounds in Whole Blood for Veterinary Use. Int. J. Mol. Sci..

[15] Ramspek C.L., Jager K.J., Dekker F.W., Zoccali C., van Diepen M. (2021). External validation of prognostic models: What, why, how, when and where?. Clin. Kidney J..

[16] Collins G.S., Dhiman P., Ma J., Schlussel M.M., Archer L., Van Calster B., Harrell F.E., Martin G.P., Moons K.G.M., van Smeden M. (2024). Evaluation of clinical prediction models (part 1): From development to external validation. BMJ.

[17] Adin D., Freeman L., Stepien R., Rush J.E., Tjostheim S., Kellihan H., Aherne M., Vereb M., Goldberg R. (2021). Effect of type of diet on blood and plasma taurine concentrations, cardiac biomarkers, and echocardiograms in 4 dog breeds. J. Vet. Intern. Med..

[18] Takita H., Kabata D., Walston S.L., Tatekawa H., Saito K., Tsujimoto Y., Miki Y., Ueda D. (2025). A systematic review and meta-analysis of diagnostic performance comparison between generative AI and physicians. NPJ Digit. Med..

[19] Jermy B., Läll K., Wolford B.N., Wang Y., Zguro K., Cheng Y., Kanai M., Kanoni S., Yang Z., Hartonen T. (2024). A unified framework for estimating country-specific cumulative incidence for 18 diseases stratified by polygenic risk. Nat. Commun..

[20] Chaturvedi M., Köster D., Bossuyt P.M., Gerke O., Jurke A., Kretzschmar M.E., Lütgehetmann M., Mikolajczyk R., Reitsma J.B., Schneiderhan-Marra N. (2024). A unified framework for diagnostic test development and evaluation during outbreaks of emerging infections. Commun. Med..

[21] Steyerberg E.W., Harrell F.E. (2016). Prediction models need appropriate internal, internal-external, and external validation. J. Clin. Epidemiol..

[22] Saito T., Rehmsmeier M. (2015). The precision-recall plot is more informative than the ROC plot when evaluating binary classifiers on imbalanced datasets. PLoS ONE.

[23] Schmidt K., Podmore I. (2015). Current Challenges in Volatile Organic Compounds Analysis as Potential Biomarkers of Cancer. J. Biomark..

[24] Remmer H., Hintze T., Frank H., Müh-Zange M. (1984). Cytochrome P-450 oxidation of alkanes originating as scission products during lipid peroxidation. Xenobiotica.

[25] Ratcliffe N., Wieczorek T., Drabińska N., Gould O., Osborne A., Costello B.P.J.d.L. (2020). A mechanistic study and review of volatile products from peroxidation of unsaturated fatty acids: An aid to understanding the origins of volatile organic compounds from the human body. J. Breath. Res..

[26] Floss M.A., Fink T., Maurer F., Volk T., Kreuer S., Müller-Wirtz L.M. (2022). Exhaled Aldehydes as Biomarkers for Lung Diseases: A Narrative Review. Molecules.

