# Supplementary material for: HS-GC/MS-Based Targeted Blood VOC Panel for Canine Cancer Screening Using an Integrated Classification Framework in Retrospective Korea and Thailand Cohorts
Source: Int J Mol Sci. 2026 Sep 9;27(18):8016. doi: 10.3390/ijms27188016 (PMC13606845; doi:10.3390/ijms27188016)
Supplement: Supplementary file 1 [file ijms-27-08016-s001.zip › ijms-4498559-supplementary.pdf]

Table S1. Instrumental conditions and SIM parameters for HS-GC/MS analysis.

Parameter	Agilent Technologies system	Thermo Fisher Scientific system
GC	8890	Trace 1610
Mass spectrometer	5977C	ISQ 7610
Autosampler	PAL RSI 85 (Series 2)	Triplus 500 headspace
Column	TG-624SilMS capillary column (0.32 mm i.d. × 30 m, 0.18 µm film thickness)	
Oven program	Rate 1: from 50 °C (5 min) to 100 °C at 10 °C/min<br>Rate 2: from 100 °C to 120 °C at 20 °C/min<br>Rate 3: from 120 °C to 260 °C (4.33 min) at 30 °C/min	
Inlet Temp. (°C)	250	
Septum Purge Flow (mL/min)	7	
Incubation Temp. (°C)	99	
Incubation Time (min)	40	
MS Transfer Line Temp. (°C)	200	
Ion Source Temp. (°C)	260	
Carrier gas	Helium, 2 mL/min	
Ionization	Electron ionization (EI), positive ion mode	
Acquisition mode	Selected ion monitoring (SIM)	
Injection mode	Split mode, Split ratio 20 : 1	
**Compound**	**Quantifier ion (*m/z*)**	**Qualifier ion (*m/z*)**
Benzene	78	77
Toluene	91	92
Ethylbenzene	91	106
Xylene	91	106
Styrene	104	78
Undecane	57	43
Dodecane	71	57
Tridecane	57	71
Tetradecane	57	71

Table S2. Baseline characteristics of the study cohort according to class and national cohort.

Characteristic	Overall	Cancer	Non-cancer	*p*-value*	Korea	Thailand	*p*-value†
Individuals, n	590	167	423		321	269	
**Age**				**< 0.001**			**< 0.001**
Mean ± SD, years	8.1 ± 4.4	10.6 ± 3.4	7.2 ± 4.3		8.7 ± 4.5	7.4 ± 4.2	
Median (IQR), years	8.0 (4.0-11.8)	11.0 (8.0-13.0)	7.0 (3.0-11.0)		9.0 (5.0-12.0)	7.0 (4.0-10.0)	
Range, years	1-18	2-18	1-17		1-18	1-17	
**Biological sex, n (%)**				**0.875**			**0.270**
Male	321 (54.4)	90 (53.9)	231 (54.6)		168 (52.3)	153 (56.9)	
Female	269 (45.6)	77 (46.1)	192 (45.4)		153 (47.7)	116 (43.1)	
**Sex/neuter status, n (%)**				**< 0.001**			**< 0.001**
Intact male	119 (20.2)	35 (21.0)	84 (19.9)		22 (6.9)	97 (36.1)	
Castrated male	202 (34.2)	55 (32.9)	147 (34.8)		146 (45.5)	56 (20.8)	
Intact female	89 (15.1)	10 (6.0)	79 (18.7)		25 (7.8)	64 (23.8)	
Spayed female	180 (30.5)	67 (40.1)	113 (26.7)		128 (39.9)	52 (19.3)	

Values are presented as mean ± standard deviation (SD), median (interquartile range [IQR]), range, or n (%), as appropriate. *Comparison between cancer and non-cancer samples; †comparison between Korea and Thailand cohorts. *P*-values indicate comparisons of the overall distribution of each characteristic and not category-specific comparisons. Age was compared using the Mann–Whitney U test, and biological sex and sex/neuter status were compared using the chi-square test. No missing values were present for age or sex/neuter status. Baseline characteristics were not used for model input, data partitioning, or covariate-adjusted performance estimation.

Table S3. Performance of the integrated classification framework in dogs aged ≥8 years.

Performance metric	Mean ± SD	95 % CI	Min-max range
AUROC	0.911 ± 0.029	0.900 – 0.921	0.852 – 0.964
AUPRC	0.909 ± 0.027	0.899 – 0.920	0.840 – 0.964
Balanced accuracy	0.844 ± 0.032	0.832 – 0.856	0.775 – 0.914
Accuracy	0.861 ± 0.030	0.849 – 0.872	0.796 – 0.924
Sensitivity	0.763 ± 0.055	0.743 – 0.784	0.650 – 0.860
Specificity	0.925 ± 0.038	0.910 – 0.939	0.844 – 0.984
Precision / PPV	0.873 ± 0.057	0.852 – 0.894	0.735 – 0.971
NPV	0.856 ± 0.032	0.844 – 0.867	0.806 – 0.910
F1-score	0.813 ± 0.041	0.797 – 0.828	0.714 – 0.902
MCC	0.708 ± 0.065	0.684 – 0.732	0.556 – 0.843
Cohen's κ	0.702 ± 0.064	0.678 – 0.726	0.556 – 0.840
